# Health Care Content and Engagement in Chronic Illness Instagram Posts: Content Analysis

**DOI:** 10.2196/57523

**Published:** 2025-09-26

**Authors:** Lindsey D Daon, Fu-Shiuan Whitney Lee, Olga Saynina, C Jason Wang

**Affiliations:** 1 Division of General Pediatrics and Adolescent Medicine, Department of Pediatrics and Adolescent Medicine, Mayo Clinic, 200 First Street SW, Rochester, MN, 55905, United States, 1 5072936631; 2 Department of Pediatrics, Stanford University School of Medicine, Stanford, CA, United States; 3 Department of Health Policy, Stanford University, Stanford, CA, United States

**Keywords:** social media, chronic illness, content analysis, Instagram, infodemics, web-based communities, digital health, social presentation theory, social cognitive theory, illness identity theory

## Abstract

**Background:**

Instagram and other social media platforms provide a unique environment for people with chronic illnesses to share experiences, but posts with higher engagement may also shape behavior. The hashtag #ChronicIllness appears in over 5 million posts, reflecting the large digital community where users seek validation, connection, and support. Frameworks such as social cognitive theory, self-presentation theory, and illness identity theory suggest that highly engaging content can shift social norms and drive behavior change via observational learning. Despite the strong theoretical basis for this behavioral impact, little is known about what chronic illness–related content is the most engaging.

**Objective:**

The aim of this study is to identify the content of Instagram posts related to chronic illness and determine if health care content is associated with higher engagement.

**Methods:**

This study is a mixed methods content analysis of 279 publicly available Instagram posts tagged with #chronicillness, #chronicallyill, or #spoonie. Posts were selected via convenience sampling and included if they featured original, nonvideo content. Photos, hashtags, and captions were coded for themes including location, medical equipment, health care experience, and illness identity. Quantitative metrics, such as likes, comments, and overperforming scores (a normalized metric of engagement), were extracted using CrowdTangle. Multivariate analyses assessed if health care content (posts featuring health care experiences or photos in a medical setting or with medical equipment) was associated with a higher odds of overperforming.

**Results:**

Posts had a median of 25 (IQR 0-14,936) likes, 3 (IQR 0-525) comments, and 20 (IQR 1-31) hashtags. A total of 222 (80%) posts were created by women, and 110 (40%) were overperforming. Photo analysis (260 posts with 406 photos) showed 27 (10%) in health care settings, and 49 (19%) included medical equipment, with 10 (4%) featuring invasive devices (eg, intravenous lines and feeding tubes), which were strongly associated with higher engagement. Hashtag analysis revealed that 243 (87%) posts referenced a medical condition, most commonly chronic pain (n=101, 36%), fibromyalgia (n=56, 20%), and Ehlers-Danlos syndrome (n=38, 14%), while 57 (20%) included medical interventions. Captions reflected 4 main themes: medical experience, illness journey, connection, and nonillness experiences. In multivariate regression analysis, longer captions (odds ratio [OR] 2.44, 95% CI 1.05‐5.67), health care content (OR 1.85, 95% CI 1.00‐3.42), and invasive medical equipment (OR 6.19, 95% CI 1.16‐32.99) were independently associated with overperforming.

**Conclusions:**

Posts featuring health care content and invasive medical equipment were associated with significantly more engagement, suggesting that medicalized portrayals of illness may be amplified on Instagram. This visibility may offer support but also risks reinforcing illness-centered identities and overmedicalization through the influence of observational learning and identity formation. Medical professionals must be aware of these trends and promote balanced, evidence-based content. Future research should explore how social media shapes health behaviors, identity, and utilization to mitigate potential harms while preserving support.

## Introduction

#Chronicillness is trending on social media, with over 5 million posts on Instagram alone [1]. Social media platforms, like Instagram, TikTok, and Facebook, serve as a space for individuals to share personal health experiences, connect with others, and seek validation. While these platforms offer support and self-empowerment opportunities, they also shape how chronic illness is perceived and presented, reinforcing certain narratives over others and influencing illness identity and behaviors. Three theoretical frameworks can be used to understand social media’s influence on behavior: social cognitive theory (SCT), self-presentation theory (SPT), and illness identity theory (IIT).

For viewers of chronic illness content, SCT describes how social media serves as a catalyst for behavior change. This occurs through reciprocal determinism, the interaction between personal characteristics, behavioral patterns, and environmental influences, particularly through observational learning [2]. This process, which involves attention, retention, reproduction, and reinforcement, shapes how viewers engage with health-related digital content [3]. Social media users span all age groups, with many individuals turning to these platforms for health-related information [4-6]. As web-based health communities grow, they provide both valuable peer support and potential pathways for symptom reinforcement and behavior change [7-9]. Ziebland and Wyke [10] raise concerns that the information people learn from social media could be used to overexaggerate symptoms to manipulate health care encounters, underscoring the need to understand the content shared on social media and its potential influence on health behavior.

For content creators, SPT can be used to understand how social media influences their behavior by shaping the way they curate and share their experiences. SPT explains how individuals attempt to control their public image through the selective presentation of behaviors, appearance, and communication [11]. Social media amplifies this effect as creators can add, remove, or edit content at will. This allows creators to present themselves in ways that maximize engagement and positive feedback, reinforcing certain narratives over others [12]. This narrative reinforcement can have cascading effects, as Masur et al [13] found that repeated exposure to behaviors on social media can change perceived norms and influence social media viewers’ self-disclosure patterns as well.

Both frameworks help explain how both creators and viewers may become more deeply embedded in an illness identity. According to IIT, chronic illness can become a core part of one’s self-concept, behavior, and self-perception across four dimensions [14]. The dimensions of acceptance and enrichment are associated with better health outcomes, while rejection and engulfment are associated with greater distress and maladaptive coping [15,16]. A study by Gabarron et al [17] highlights the concern about social media’s influence on illness identity, finding that users are least engaged with content that fosters empowerment, such as health education and research, and most with personal narratives. This suggests that users are more drawn to relatable, emotionally compelling content that may reinforce illness-centered narratives rather than recovery-oriented perspectives. The content of such narratives becomes important in the context of social media algorithms, which boost the most engaging content, further shaping chronic illness narratives [18].

The emergence of illness-centered digital identities is not new. In 2013, the term “Spoonie” was popularized through a viral blog post, becoming a widely recognized identity for those with chronic conditions [19]. While this sense of community can be empowering, it also raises concerns about the reinforcement of illness identity. Media reports have highlighted cases in which individuals exaggerate symptoms or seek unnecessary medical interventions in pursuit of validation [20,21]. Beyond anecdotal reports, research has documented the psychogenic spread of illness through social media, such as with functional tics and dissociative identity disorder, where symptoms increased among social media users following viral exposure [22,23]. Similarly, studies have found a rise in self-diagnosis trends for mental health conditions [24,25] and the digital amplification of various symptoms [26-28].

With these rising concerns, it is essential to examine the types of chronic illness–related content shared on web-based platforms and which narratives receive the most attention, represented by user engagement. Attention is a key driver of observational learning and self-presentation, reinforcing certain behaviors and narratives. Highly visible content is more likely to be seen, remembered, replicated, and incorporated into one’s identity. Understanding existing content and its engagement patterns is crucial to recognizing the behaviors that people who self-identify as having chronic illness are exposed to and may adopt. This study aims to analyze Instagram posts related to chronic illness, examining both the content being posted and that which receives the most user engagement.

## Methods

### Study Design

**Figure 1.**
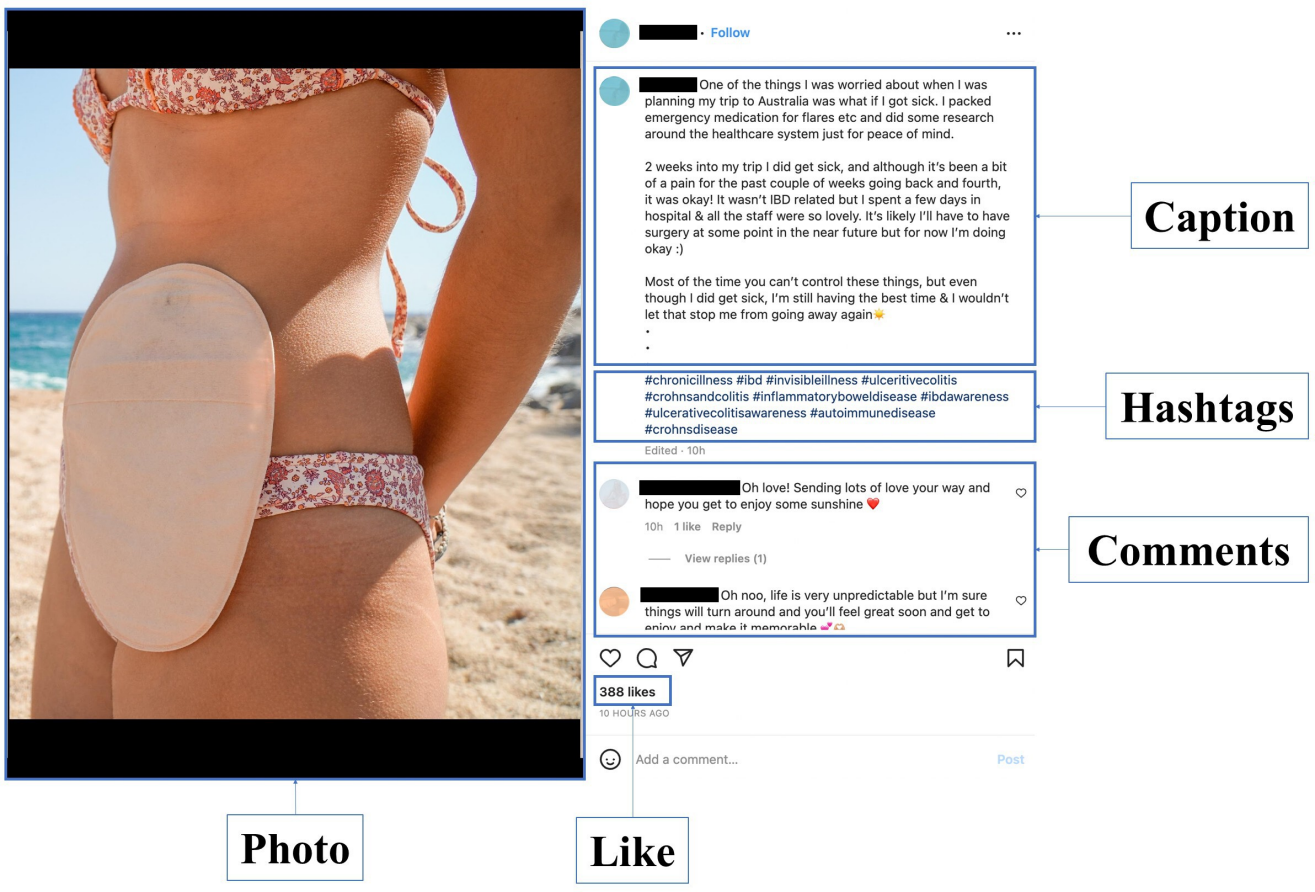
Recreated Instagram post using a stock photo, with the key components labeled (eg, caption, hashtags, photo, likes, and comments).

This was a mixed methods study, combining qualitative and quantitative methods, to conduct a multimodal content analysis of Instagram posts. The analysis involved the qualitative examination of photos, hashtags, captions, and quantitative metrics, such as overperforming scores, likes, comments, and followers. Figure 1 provides a visual representation of the components of an Instagram post.

### Sampling and Data Collection

The units of analysis for this study are Instagram posts, which were selected using convenience sampling. Convenience sampling was necessary due to the large volume of Instagram posts and the desire to focus on more recent posts as content on social media changes over time. Three search terms, that is, hashtags, were selected for their relevance to individuals with chronic illness, their broad representation of chronic illness rather than specific diseases, and the substantial number of posts associated with each hashtag: #chronicillness, #chronicallyill, and #spoonie.

To minimize the influence of algorithms on postselection, two independent reviewers (LDD and FSWL) conducted separate searches on an arbitrarily chosen date (October 26, 2021) and chose qualifying posts from the “Most Recent” section of the search results. Posts were included if they were publicly available and appeared to be original content, such as photos, artwork, or products being promoted. Posts were excluded if they contained videos or the photos were nonoriginal content, such as memes or reposted content from another person’s account. Each reviewer selected posts in reverse chronological order until 100 posts for each hashtag were included, resulting in a total of 300 posts per reviewer. The reviewers cross-compared the selected posts to ensure that only common posts were included, removed duplicates, and resolved inclusion or exclusion inconsistencies, resulting in a final sample of 279 posts.

The data for analysis, including caption text, hashtags, number of likes, and overperforming score, were downloaded from CrowdTangle and cleaned using Excel (Microsoft Corp), 10 days after identifying the posts. This 10-day delay allowed the posts to receive views, the bulk of which occurred within the first few days after being posted. During data cleaning, the number of followers and country location for each account were verified by reviewing the associated account profiles and posts. These data were used for the descriptive statistics (eg, likes, comments, total interactions, followers, and number of photos) for all 279 posts. Photos were not included in the CSV file and were analyzed directly on the CrowdTangle website in March 2022. By the time photo analysis occurred, 19 posts had been removed or made private; thus, only 260 posts underwent photo analysis and inclusion in the multivariate analysis.

### Qualitative Analysis

The qualitative analysis involved multimodal content analysis of Instagram posts, including photos, hashtags, and captions. Multiple elements of the posts were used to determine gender and identify the presence of advertising content. Gender identification involved visually inspecting the photos and assessing the captions or hashtags for gender-specific language. Advertising content was defined as any promotional material related to businesses or products that have the potential to generate income, such as product shops, coaching services, podcasts, or blogs. Two coders (LDD and FSWL) collaborated to analyze the content of the posts.

Photos for each post underwent visual content analysis simultaneously by the two coders. Each photo was coded for location, presence of medical equipment, presence of a support animal, and number of people. Location was coded by medical (eg, hospital and clinic) or nonmedical (eg, yard, neighborhood, and house). Medical equipment was coded by invasive (eg, intravenous lines [IVs], peripherally inserted central catheters [PICCs], and feeding tubes) or noninvasive (eg, medications, nasal cannula, and mobility aids). When multiple photos were present in a post, if any photo within a post contained the theme of interest, the entire post was considered to have that theme for analysis purposes.

Hashtags underwent quantitative content analysis. They were coded for mentions of specific conditions and medical interventions (Table 1). A hashtag was considered a condition if it included a specific diagnosis (eg, chronic fatigue syndrome) but not a general symptom (eg, fatigue). If a hashtag could be considered either a diagnosis or a symptom (eg, anemia, headaches, gastroparesis, and anxiety), it was coded as a diagnosis. Medical interventions included procedures, testing, and treatments.

**Table 1.** Summary of key findings from photo and hashtag content analysis^a^.

	Value, n (%)
Photo analysis (n=260)	
Location of photo	
Health care setting (ie, clinic or hospital)	27 (10.4)
Non–health care setting (ie, home and outdoors^b^)	199 (76.5)
Location indeterminate (ie, art and slides)	34 (13.1)
Medical equipment	49 (18.8)
Invasive medical equipment^c^	10 (3.8)
Noninvasive medical equipment^d^	39 (15)
Hashtag analysis (n=279)	
Condition^e^	243 (87.1)
Chronic pain	101 (36.2)
Fibromyalgia	56 (20.1)
Autoimmunity	38 (13.6)
Ehlers-Danlos syndrome (EDS)	38 (13.6)
Postural orthostatic tachycardia syndrome (POTS)	36 (12.9)
Anxiety	31 (11.1)
Myalgic encephalitis/chronic fatigue syndrome (ME/CFS)	27 (9.6)
Dysautonomia	25 (9)
Depression	22 (7.9)
Endometriosis or adenomyosis	21 (7.5)
Gastroparesis	20 (7.2)
Inflammatory bowel disease (IBD)	20 (7.2)
Arthritis	15 (5.4)
Headache	14 (5)
Lupus	13 (4.7)
Multiple sclerosis (MS)	12 (4.3)
Post traumatic stress disorder (PTSD)	11 (3.9)
Lyme disease	10 (3.6)
Medical Intervention	57 (20.4)
Mobility aids	25 (9)
Medications	16 (5.7)
Invasive procedures	15 (5.4)
Feeding tubes	11 (3.9)

a Photos from 260 posts were included in the analysis. Hashtags from all 279 posts were included in the analysis.

b Including nature, yards, neighborhoods, and cities.

c Including peripheral intravenous (PIV) lines, peripherally inserted central catheter (PICC) lines, ports, and feeding tubes.

d Including medications, bandages, blood vials, mobility aids (canes, wheelchairs, walkers), compression socks, hospital beds, hospital gowns, telemetry wires, nasal cannulas, oxygen tanks, diagnostic images, and visual symptoms of illness.

e 107 different conditions were identified. Conditions that were mentioned in 10 or more posts are listed.

**Figure 2.**
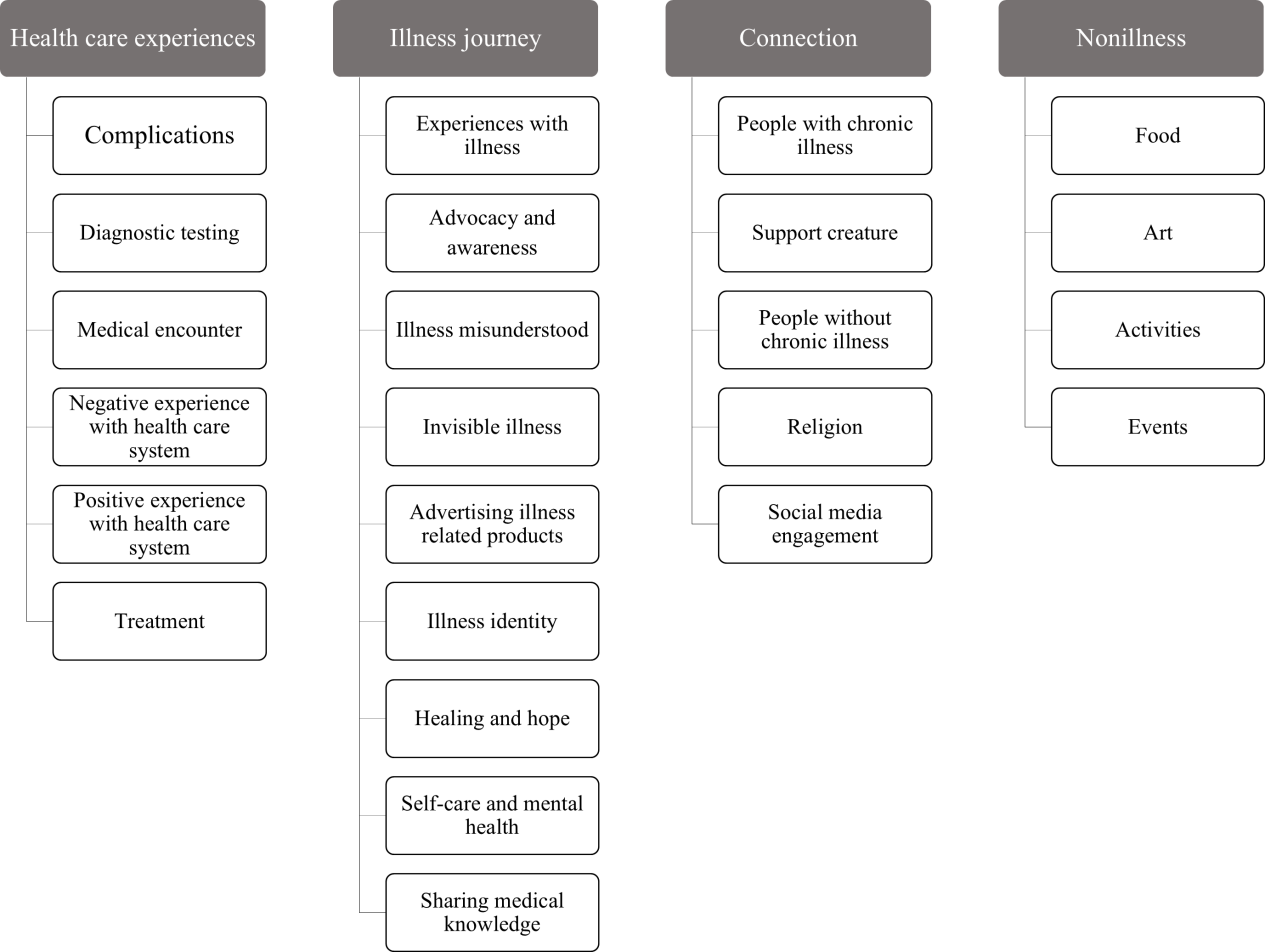
The post captions were independently coded by 2 reviewers using an iterative and inductive process, resulting in 4 major themes with associated subthemes. Coding discrepancies were discussed and resolved through consensus.

Captions underwent content analysis using an iterative and inductive process. The initial categories and codes were based on a first pass review of the included post captions, illness identity framework, and the research question, and included health care–related, disease-related, personal feelings, interactions with others, life events or beliefs, and illness identity. The two reviewers then simultaneously coded the first 20 posts to ensure mutual agreement and understanding of the categories and emerging codes. The remaining posts were divided between the two team members for independent coding. As new codes emerged or at least every 50 posts, the reviewers met frequently to review each other’s work, refine categories and codes, and resolve inconsistencies. If a consensus was unable to be reached, a third team member (CJW) was used as a tiebreaker. The final list of categories and codes can be seen in Figure 2.

### Quantitative Analysis

The overperforming score [29], calculated by CrowdTangle, is a metric that compares a post’s engagement (likes, comments, and shares) to posts from the same account. This allows for normalization of engagement across accounts, reducing the advantage of accounts with more followers or frequent posts. A score greater than 1 indicates overperformance, a score less than 0 suggests underperformance, and scores between 0 and 1 are considered indeterminate. Posts that overperform suggest higher than typical engagement on that post, highlighting content that resonates more with audiences.

For this study, overperforming was used as a proxy for user engagement, as it accounts for both absolute engagement and relative performance within an account’s posting history. This approach helps mitigate the effects of larger accounts naturally receiving more engagement and allows for a more meaningful comparison of content performance.

The overperforming score was used as the primary dependent variable to investigate the association between health care content and engagement. Health care content is a summary variable that counted posts that included photos with medical equipment (ie, peripherally inserted central catheter lines, feeding tubes, IVs, mobility assistive devices, and medications), medical location (ie, hospital and clinic), or a description of a specific health care encounter (ie, clinic visit, emergency department, and starting a new medication).

Bivariate and multivariate models were conducted by the data analyst (OS) to examine the dependent variable (overperforming score) in relation to several independent variables, outlined in Table 2. For the multivariate analysis, the overperforming score was transformed into a binary variable, categorizing a score of 1 or greater as “overperforming” and scores less than 1 as “not overperforming.”

**Table 2.** OR^a^ bivariate and multivariate analyses.

Variable	Bivariate, OR (95% CI)	Model 1, OR (95% CI)	Model 2, OR (95% CI)	Model 3, OR (95% CI)
People present in photo	1.511 (0.9-2.537)	1.208 (0.655-2.227)	1.181 (0.645-2.162)	1.195 (0.647-2.207)
Type of post (photo vs album)	1.653 (0.957-2.856)	1.47 (0.763-2.831)	1.496 (0.778-2.875)	1.315 (0.673-2.566)
Hashtag: quartile 2 versus 1	1.333 (0.68-2.612)	1.527 (0.663-3.518)	1.503 (0.655-3.448)	1.874 (0.789-4.446)
Hashtag: quartile 3 versus 1	1.379 (0.706-2.693)	2.156 (0.918-5.066)	1.955 (0.844-4.528)	2.296 (0.961-5.484)
Hashtag: Quartile 4 versus 1	1.24 (0.62-2.481)	1.136 (0.464-2.782)	1.055 (0.435-2.558)	1.24 (0.494-3.113)
Caption length: quartile 2 versus 1	1.304 (0.637-2.668)	1.186 (0.496-2.836)	1.167 (0.493-2.767)	1.137 (0.475-2.721)
Caption length: quartile 3 versus 1	1.875 (0.929-3.783)	2.048 (0.868-4.829)	2.112 (0.902-4.948)	2.145 (0.909-5.064)
Caption length: quartile 4 versus 1	2.727 (1.352-5.499)^b^	2.02 (0.846-4.824)	2.438 (1.047-5.674)^b^	2.179 (0.925-5.135)
Number of condition: 3‐4 versus 1‐2	0.874 (0.479-1.596)	0.591 (0.288-1.211)	0.655 (0.324-1.325)	0.628 (0.307-1.284)
Number of condition: 5+ versus 1‐2	0.876 (0.451-1.703)	0.646 (0.298-1.404)	0.748 (0.352-1.591)	0.731 (0.34-1.571)
Gender: male versus female	0.267 (0.057-1.249)	0.292 (0.057-1.502)	0.27 (0.054-1.354)	0.279 (0.056-1.391)
Gender: other versus female	0.543 (0.27-1.091)	0.404 (0.127-1.285)	0.39 (0.123-1.239)	0.425 (0.134-1.345)
Number of followers: quartile 2 versus 1	1.061 (0.541-2.083)	0.955 (0.416-2.191)	0.983 (0.432-2.236)	1.051 (0.456-2.422)
Number of followers: quartile 3 versus 1	0.942 (0.478-1.856)	0.852 (0.366-1.984)	0.813 (0.352-1.88)	0.868 (0.371-2.034)
Number of followers: quartile 4 versus 1	0.907 (0.458-1.795)	0.741 (0.309-1.781)	0.727 (0.305-1.733)	0.788 (0.327-1.899)
Health care content summary^c^	2.025 (1.224-3.349)^b^	1.854 (1.004-3.421)^b^	—^d^	—
Photo with noninvasive medical equipment	1.213 (0.609-2.414)	—	1.005 (0.474-2.134)^b^	—
Photo with invasive medical equipment	6.526 (1.357-31.382)^b^	—	—	6.187 (1.16-32.991)^b^
Photo in medical location	1.248 (0.559-2.788)	—	—	—
Medical experience	1.831 (1.078-3.11)^b^	—	—	—
Condition discussed	1.737 (0.977-3.091)	—	—	—
Support object	0.697 (0.324-1.498)	—	—	—
Nonillness content^e^	0.664 (0.393-1.121)	—	—	—
Advertisement	0.7 (0.37-1.323)	—	—	—

a OR: odds ratio.

b Statistically significant odds ratio with 95% CI.

c Summary variable that includes photo with medical equipment (invasive or noninvasive), photo in a medical location, or a caption containing the category Health Care Experience.

d Indicates a controlled-for variable in each model.

e Nonillness content if photo or caption did not include illness journey or health care content.

### Ethical Considerations

Per Stanford University’s institutional review board, this study, which examines publicly available Instagram posts, was exempt from the institutional review board approval and informed consent as it does not meet the criteria for human participant research. No direct interaction with users occurred, and all data were publicly accessible and deidentified during the cleaning process for quantitative analysis. While photos could not be fully deidentified due to their role in visual coding, all coded data were anonymized and stored without user identifiers. To protect privacy in visual presentation, all illustrative images used in figures were recreated using licensed Shutterstock photos and adapted to mimic the structure and content of actual posts; no original user photos were published.

## Results

### Post Characteristics

**Figure 3.**
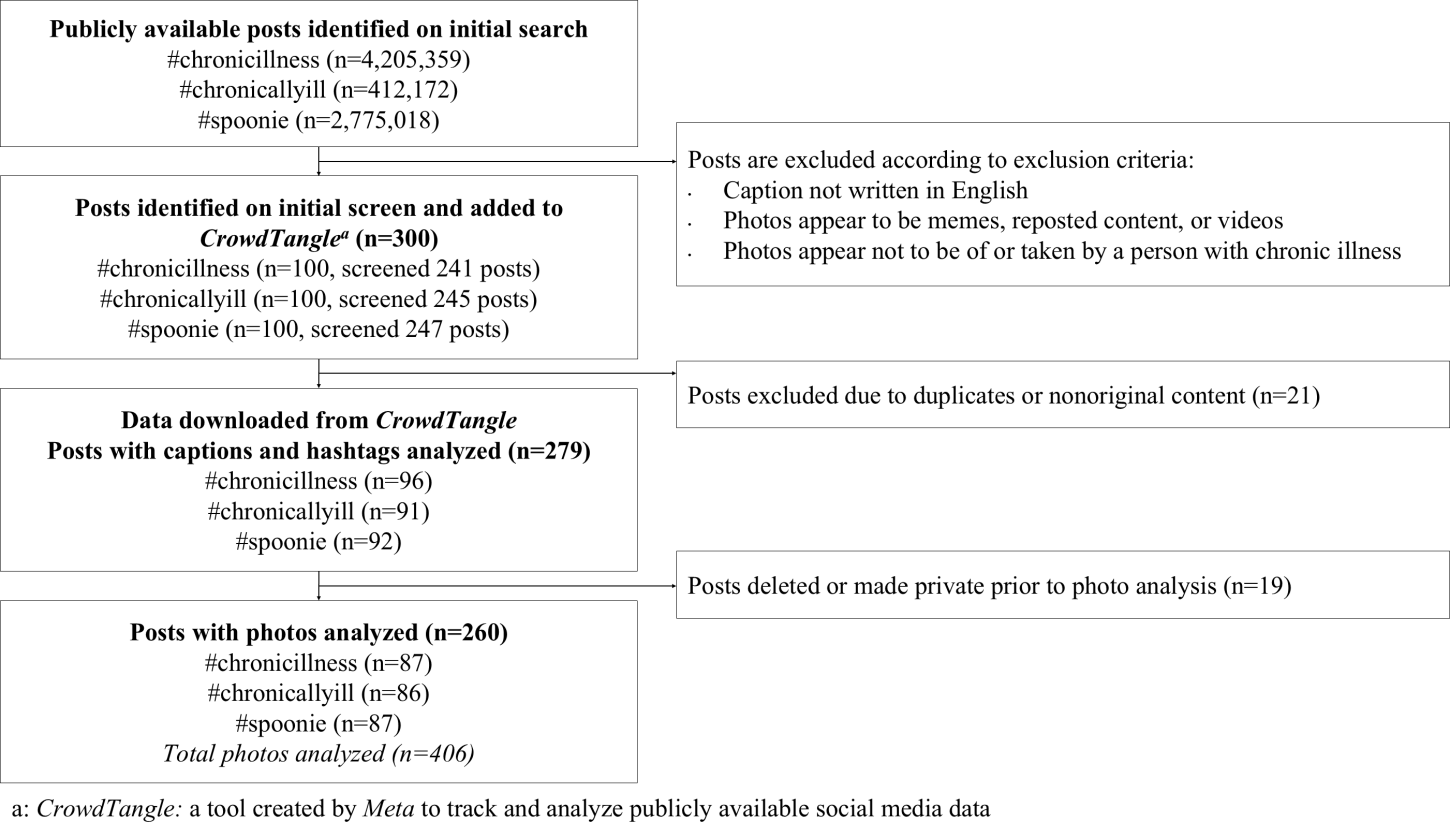
Post selection process flowchart. An initial search of the 3 hashtags on October 16, 2021, revealed millions of posts per search term. The two reviewers used convenience sampling, from newest to oldest, to select posts for inclusion, resulting in a total of 300 posts per reviewer. After cross-comparison, duplicate removal, and exclusion resolution, 279 posts were included in the final dataset. Photos were analyzed separately in March 2022, with 260 posts available at that time for visual analysis and inclusion in multivariate analysis.

A total of 279 posts were included in the descriptive statistics analysis (Table 3) and the caption and hashtag content analysis. Among these, only 260 posts containing 406 photos were used for the visual analysis and multivariate analysis (Figure 3). The median number of followers at the time of data collection was 661 (IQR 326-1624). Posts received a median of 25 (IQR 12-53) likes and 3 (IQR 1-8) comments. The median number of hashtags per post was 20 (IQR 12-28). Of the analyzed posts, 110 (40%) were considered overperforming, and 52 (20%) contained marketing content. Most posts were authored by women (n=222, 80%) and featured a single photo (n=209, 75%). Age information was unavailable.

**Table 3.** Characteristics of the 279 posts included in the analysis, summarizing the metadata, creator demographics, and content features.

Characteristic of post	Value (n=279)
Hashtags (total), median (IQR)	20 (12-28)
Caption length (in characters), median (IQR)	596 (396-1053)
Conditions (total in hashtags), median (IQR)	2 (1-4)
Followers (total), median (IQR)	661 (326–1624)
Likes (total), median (IQR)	25 (12-53)
Comments (total), median (IQR)	3 (1-8)
Interactions (total), median (IQR)	29 (14-63)
Gender, n (%)	
Women	222 (79.6)
Men	12 (4.3)
Other or unknown	45 (16.1)
Country of post, n (%)	
United States	51 (18.2)
United Kingdom	16 (5.7)
Others^a^	18 (6.4)
Unknown	194 (69.5)
Content features, n (%)	
Overperforming status	110 (39.4)
Health care content present	97 (34.7)
Advertising present^b^	52 (18.6)
Type of post—album^c^	70 (25.1)

a Canada, Mexico, Ireland, Sweden, the Netherlands, South Africa, India, Australia, and New Zealand.

b Advertisements included podcasts, blogs, supplements, hyperbaric oxygen therapy, health coaching services, money donation accounts, and products from digital shops such as “tubie” supplies and chronic illness pins or stickers or graphic tees or books.

c Albums are posts that contain multiple photos.

### Qualitative Analysis

#### Photos

Out of the 260 posts with photos analyzed (Table 1), 27 (10%) were in a health care setting, and 49 (19%) contained medical equipment. Among the posts featuring medical equipment, 10 posts depicted invasive devices, primarily IVs and feeding tubes, while 39 posts showed noninvasive equipment, mainly mobility aids. Support animals, such as dogs, cats, and stuffed animals, were present in 34 (13%) posts, often with captions written from the perspective of the support animal. The number of people in the photos ranged from 0 to 5, with a median of 1 (IQR 0-1).

#### Hashtags

A total of 5454 hashtags across the 279 posts were analyzed for conditions and medical interventions (Table 1, conditions and interventions mentioned in 10 or more posts; Table S1 in Multimedia Appendix 1, full list of hashtagged conditions and interventions). Among the analyzed posts, 243 (87%) posts contained hashtags referencing specific medical conditions, with 107 different conditions identified.

Many conditions are those that have a susceptibility to self-diagnosis, including fibromyalgia (56 posts) [30], anxiety (31 posts) [25], depression (22 posts) [31], Lyme disease (10 posts) [32], mast cell activation syndrome (5 posts) [33], and dissociative identity disorder (3 posts) [24]. Other frequently mentioned conditions are functional disorders such as chronic pain (101 posts), myalgic encephalitis/chronic fatigue syndrome (27 posts), headache (14 posts), and irritable bowel syndrome (7 posts) [34]. In contrast, it was uncommon to see posts referring to conditions that are less likely to be self-diagnosed or functional, such as inflammatory bowel disease (20 posts), multiple sclerosis (12 posts), diabetes (8 posts), and cancer (6 posts). Additionally, 57 (20%) posts included hashtags referencing medical interventions, with 28 different interventions identified.

#### Captions

Content analysis of the captions revealed four major themes: (1) health care experiences (n=97, 35%), (2) illness journey (n=182, 65%), (3) connection (n=155, 55%), and (4) nonillness experiences (n=91, 33%; Table 4). The health care experiences theme encompassed content related to specific health care encounters, diagnostic testing, and medical treatments. The illness journey theme explored the impact of chronic illness on daily life, including the ways in which other people misunderstood their conditions, the concept of “invisible illness,” and the promotion of products related to their illnesses. Invisible illness is a phrase used to raise awareness and highlight the unique struggles faced by individuals whose conditions may not be immediately apparent to others. The connection theme highlighted relationships established among individuals with chronic illnesses, the support received from both human and nonhuman sources, and advice for those without chronic illnesses to foster empathy and understanding. Finally, the nonillness experiences theme includes food, art, and activities. Excerpts from the full list of categories and codes are provided in Table S2 in Multimedia Appendix 1. An in-depth exploration of the qualitative codes and representative quotes will be the focus of future work.

**Table 4.** Summary of the 4 major caption content themes with the number of posts that included the theme (the same post may include multiple themes).

Caption theme	Posts, n (%)	Caption excerpt
Illness journey	182 (65)	“I can pretend I’m ‘healthy’ but I know deep down, that it’s all fake. I have a pretty negative self image. I see a broken person. I struggle with exhaustion so heavy I feel drunk tired.”
Connection	155 (55)	“Thank you to everyone who checked in with me these past 2 months. You have no idea how much I appreciate it!”
Health care experience	97 (35)	“Throw back to when I had my PICC Line placed in my arm 3 years ago. I sure don’t miss having a PICC Line... This was when my illnesses became much more visible... Having a tube in my arm for 7.5 months says a lot about my health. I’m glad I had a port surgically implanted 2 years & 5 months ago & have been PICC Line free for 2 years & 5 months now…”
Nonillness experience	91 (33)	“Spent yesterday decorating for Halloween! Can’t wait to show you all”

### Quantitative Analysis

**Figure 4.**
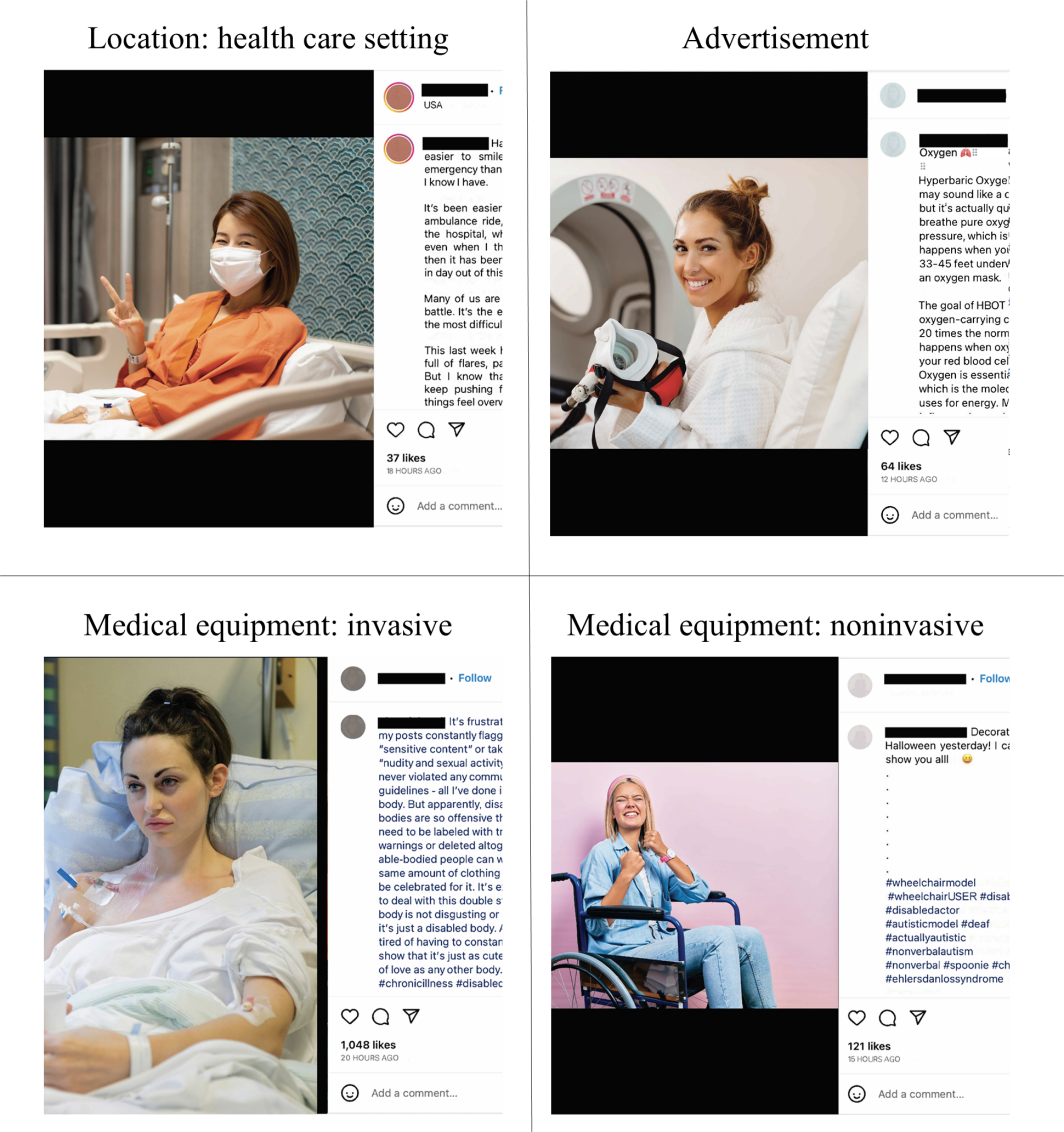
Example posts created from Shutterstock images of visual themes identified during photo content analysis, including health care setting, invasive medical equipment, noninvasive medical equipment, and advertisements.

The bivariate logistic regression (Table 2) showed that posts had a statistically significantly higher odds of overperforming, or receiving more user engagement if they contained a longer caption (odds ratio [OR] 2.727, 95% CI 1.352, 5.499), health care content (OR 2.025, 95% CI 1.224-3.349), a photo with invasive medical equipment (OR 6.526, 95% CI 1.357-31.382), or a health care experience (OR 1.831, 95% CI 1.078-3.11). Health care content is a summary variable that includes posts with a photo of medical equipment, a photo in a medical location, or a caption containing the theme health care experience. During multivariate regression analysis, the calculations were controlled for the presence of people in the photo, type of post, number of hashtags, caption length, number of conditions hashtagged, gender, and number of followers. After controlling for these variables, caption length (OR 2.438, 95% CI 1.047-5.674), health care content (OR 1.854, 95% CI 1.004-3.421), and photos with invasive medical equipment (OR 6.187, 95% CI 1.16-32.991) remained statistically significant. Figure 4 provides examples of posts containing overperforming content and advertisements.

## Discussion

### Principal Findings

This study identified the content of Instagram posts related to chronic illness and assessed if health care content received higher engagement. The captions frequently included personal illness journey (n=182, 65%), while 35% (n=97) featured overt references to health care experiences. The quantitative analysis revealed that posts with longer captions (OR 2.44, 95% CI 1.05-5.67), health care content (OR 1.85, 95% CI 1.00-3.42), and images showing invasive medical equipment (OR 6.19, 95% CI 1.16-32.99) were more likely to overperform, even when controlling for other posts’ characteristics. These findings suggest that more descriptive and medicalized portrayals of illness receive greater user engagement.

These findings support prior work demonstrating that social media is used to share illness narratives and medical experiences [5,17,35]. Notably, the study adds that health care content, especially posts with images showing invasive medical equipment, garners remarkably high engagement. Medicalized portrayals of chronic illness may be amplified by social media algorithms, resulting in implications for both creators and viewers, as it may reinforce a narrow, medicalized view of chronic illness. Repeated exposure to algorithmically amplified content increases visibility and creates perceived social norms, shaping how users interact with and internalize illness-related information. Through curated self-presentation, observational learning, and identity reinforcement, social media becomes not just a space for sharing experiences, but a place for shaping illness identity and behavior [3,36,37].

For content creators, higher engagement provides positive reinforcement, encourages certain behaviors, and motivates the production of similar posts. SPT suggests that creators will curate their content based on their desired identity and feedback [12]. These results suggest posts featuring medicalized portrayals of illness receive more engagement, which may reinforce the sick role and increase focus on health care encounters and medical interventions. This not only deepens their connection to their illness but also increases their perceived self-efficacy, which can improve their confidence in managing their condition and ability to self-advocate for their health [38]. However, it can also lead them to believe they have expert knowledge about their conditions even above medical providers, as suggested by posts that downplay physician knowledge and promote commercial self-help content.

Commercial interests further complicate this dynamic. Approximately one in five posts advertised products, ranging from alternative therapies (ie, oxygen therapy and supplements) to personal Etsy shops selling illness-related merchandise. This is consistent with the broader role of social media influencers in product marketing [39] and further showcases social media’s enhancement of self-efficacy and autonomy. It can provide accessible income opportunities for individuals who may be underserved by traditional employment. However, the potential for financial gain may further incentivize creators to medicalize their digital identity and promote content that aligns with the marketable aspects of chronic illness. As a result, authentic self-expression becomes enmeshed with commercial motivation.

For viewers, social media platforms quickly tailor content based on engagement patterns, further exposing viewers to posts like those previously viewed. An investigation by the Wall Street Journal suggests that some platforms’ algorithms use individual user engagement patterns to quickly adjust post recommendations, leading viewers to less monitored and more extreme content [40]. This is concerning through the lens of SCT’s observational learning, where individuals adopt behaviors they repeatedly see and that are reinforced through social approval. Content that receives greater attention is not only more visible and memorable but is also algorithmically promoted, increasing exposure and perceived social norms. Repeated presentation of health care encounters, and even more concerningly, invasive medical equipment in overperforming posts may normalize these interventions as a required part of the chronic illness journey. This could lead to an exaggerated emphasis on the importance of medicalization in the role of illness validation, solidifying the illness identity.

From an IIT perspective, validation through post attention may solidify an illness-centered identity. IIT describes how chronic illness becomes integrated into an individual’s identity via acceptance, enrichment, rejection, and engulfment. While acceptance and enrichment have improved health outcomes, engulfment and rejection are associated with higher medical utilization and worse outcomes [14-16]. Social media has the potential to increase enrichment through advocacy and shared learning, but one study found that viewers were actually less likely to engage with enriching content [5]. Higher engagement with medicalized content may push users toward engulfment, where illness becomes a more consuming part of identity.

The focus on medicalized content and its ability to integrate illness into one’s identity is concerning for viewers, regardless of current chronic illness diagnosis. Posts often reference self-diagnosable conditions, such as chronic pain (n=101, 36.2%), anxiety (n=31, 11.1%), and chronic fatigue (n=27, 9.6%) [25,26,31]. These experiences, which are common in the general population, are highly susceptible to somatic hypervigilance, where excessive attention to bodily sensations leads to symptom amplification and perceived illness. In web-based communities, this may lead to the pursuit of popularized diagnoses, such as postural orthostatic tachycardia syndrome (POTS) or fibromyalgia, that can result in a prolonged diagnostic odyssey seeking validation. Self-efficacy narratives compound this process by encouraging individuals to trust their self-perception over the results of clinical evaluations. Viewers are told not to give up until they receive the diagnosis they believe they have, further reinforcing the cycle of symptom monitoring, medical validation-seeking, and identity reinforcement.

For both content creators and viewers, repeated engagement with chronic illness–related content can improve self-efficacy, increasing confidence in their understanding and management of illness, even when these beliefs differ from medical expertise. Clinically, this aligns with the authors’ observations of patients requesting evaluation for rare diagnoses or requesting inappropriate treatments based on social media content. Prior research confirms that social media exposure can change behavior, with Kankova et al [36] demonstrating that influencers shape health-related attitudes and behaviors in both positive and negative ways. Other studies highlight how social media influencers drive chronic illness conversations, shape public perceptions of health, and may influence health care–seeking behaviors [17,37,41].

Posts with health care content may attract more attention due to perceived authenticity, emotional appeal, and unique insight into the lived experience of chronic illness. These narratives may provide visibility and validation for individuals who feel misunderstood by doctors and healthy individuals. While social media provides invaluable support and visibility for individuals with chronic illness, it also plays a powerful role in shaping illness identity. Increased engagement with medicalized content may inadvertently shift individuals toward an illness-centered identity, where symptoms become entwined with both one’s sense of self and social belonging. Once this identity is established, the transition to wellness can be difficult, as recovery may feel like a loss of identity and community support. The benefits of connection and validation on social media must come with recognition of the potential harms of reinforcing maladaptive illness narratives.

### Limitations

There are several limitations in this study. First, data collection was conducted on a single day, which could have skewed results toward time-sensitive events such as awareness weeks. However, our goal was not to generalize findings about specific conditions, but rather to identify broader themes in chronic illness content. Second, convenience sampling was necessary due to the large volume of posts and continuous content creation. While this approach may introduce bias, this study mitigated this by using a “most recent” search strategy to ensure that included posts reflected current discussions. Third, demographic details were not available for all posts, limiting insights into the characteristics of content creators. However, the study’s primary focus was on content rather than user demographics. Finally, while private accounts and posts were inaccessible, the study captured perspectives from the digital chronic illness community that are publicly available.

### Conclusions and Future Work

This study demonstrates that social media engagement reinforces medicalized portrayals of chronic illness, particularly through overperforming content featuring health care encounters and invasive medical equipment. By integrating the SPT, SCT, and IIT frameworks, it becomes clear that creators curate content to maximize engagement, viewers internalize and normalize behaviors through observational learning, and both adapt their illness identities based on social feedback and validation. While platforms like Instagram provide validation, support, and advocacy opportunities for people with chronic illness, they also present risks of symptom amplification, overmedicalization, and the spread of psychogenic symptoms. The commercialization of chronic illness content complicates things further by blurring the line between authentic self-expression and financially motivated narratives. These, along with the anecdotal reports of patients requesting specific diagnoses or treatments based on social media content, highlight the urgent need for health care providers to be informed about digital health trends. Medical professionals actively engaging with chronic illness content is vital to ensure accurate and evidence-based information is available to counter misinformation.

These findings underscore the importance of integrating social media literacy into clinical education and underscore the need for social media platforms and policymakers to consider how engagement metrics shape public health discourse. Future research should explore the effects of chronic illness content on both individuals and at the public health level. In particular, understanding how repeated exposure to chronic illness content influences health behaviors, self-diagnosis trends, and health care utilization is of vital importance. Further examination of social media influencers’ role in shaping chronic illness narratives is also warranted. As digital platforms evolve, a balanced approach is needed, one that leverages the benefits of web-based health communities while mitigating the risks associated with misinformation, commercial influence, and illness identity reinforcement. Health care providers can play a critical role by providing accessible, evidence-based content that empowers patients to make informed decisions. Ensuring that social media remains a space for support, education, and empowerment is essential to preventing unintended consequences of chronic illness identification and overmedicalization.

## Supplementary material

10.2196/57523 Multimedia Appendix 1 Full list of hashtag conditions and interventions and full list of categories and codes with excerpts from post.

## Abbreviations

IIT: illness identity theory
IV: intravenous line
OR: odds ratio
SCT: social cognitive theory
SPT: self-presentation theory
