# Supplementary material for: Health Care Content and Engagement in Chronic Illness Instagram Posts: Content Analysis
Source: JMIR Form Res. 2025 Sep 26;9:e57523. doi: 10.2196/57523 (PMC12468905; doi:10.2196/57523)
Supplement: Multimedia Appendix 1 [file formative-v9-e57523-s001.docx]

**Table S1.** Full list of hashtag conditions and medical interventions

| **Hashtag** | **No. of Posts**  **n=279** |
| --- | --- |
| **Condition**s | 232 (83.2%) |
| **Chronic pain** | 101 (36.2%) |
| Fibromyalgia | 56 (20.1%) |
| Disability | 51 (18.3%) |
| Autoimmune | 38 (13.6%) |
| Ehlers-Danlos syndrome | 38 (13.6%) |
| Postural orthostatic tachycardia syndrome | 36 (12.9%) |
| Anxiety | 31 (11.1%) |
| Chronic fatigue syndrome | 27 (9.7%) |
| Dysautonomia | 25 (9.0%) |
| Depression | 22 (7.9%) |
| Endometriosis/Adenomyosis | 21 (7.5%) |
| Gastroparesis | 20 (7.2%) |
| Inflammatory bowel disease | 20 (7.2%) |
| Arthritis | 15 (5.4%) |
| Headaches | 14 (5.0%) |
| Lupus | 13 (4.7%) |
| Multiple Sclerosis | 12 (4.3%) |
| Rare | 12 (4.3%) |
| Post-traumatic stress disorder | 11 (3.9%) |
| Chronic Lyme disease | 10 (3.6%) |
| Diabetes | 8 (2.9%) |
| Brain injury | 7 (2.5%) |
| Hypothyroidism | 7 (2.5%) |
| Irritable bowel syndrome | 7 (2.5%) |
| Sjogren’s disease | 7 (2.5%) |
| Bipolar disorder | 6 (2.2%) |
| Cancer | 6 (2.2%) |
| Polycystic ovary syndrome | 6 (2.2%) |
| ADHD | 5 (1.8%) |
| Celiac disease | 5 (1.8%) |
| Hypermobile spectrum disorder | 5 (1.8%) |
| Mast cell activation syndrome | 5 (1.8%) |
| Scoliosis | 5 (1.8%) |
| Anemia | 4 (1.4%) |
| Autism | 4 (1.4%) |
| Functional neurologic disorder | 4 (1.4%) |
| Psoriasis | 4 (1.4%) |
| Raynaud’s syndrome | 4 (1.4%) |
| Ankylosing spondylitis | 3 (1.1%) |
| CSF leak | 3 (1.1%) |
| Dissociative identity disorder | 3 (1.1%) |
| Eczema | 3 (1.1%) |
| Hyperthyroidism | 3 (1.1%) |
| Panic disorder | 3 (1.1%) |
| Superior mesenteric artery syndrome | 3 (1.1%) |
| Undiagnosed | 3 (1.1%) |
| Asthma | 2 (0.7%) |
| Chronic kidney disease | 2 (0.7%) |
| Connective tissue disorder | 2 (0.7%) |
| Cyclic vomiting | 2 (0.7%) |
| Epilepsy | 2 (0.7%) |
| Immune deficiency | 2 (0.7%) |
| Interstitial cystitis | 2 (0.7%) |
| Intracranial hypertension | 2 (0.7%) |
| Liver disease | 2 (0.7%) |
| Long COVID | 2 (0.7%) |
| COPD | 2 (0.7%) |
| Emphysema | 2 (0.7%) |
| Gene mutation | 2 (0.7%) |
| Restless leg syndrome | 2 (0.7%) |
| Sarcoidosis | 2 (0.7%) |
| Adrenal insufficiency | 1 (0.4%) |
| Agoraphobia | 1 (0.4%) |
| Allergy | 1 (0.4%) |
| Aseptic meningitis | 1 (0.4%) |
| Avascular necrosis | 1 (0.4%) |
| Arteriovenous malformation | 1 (0.4%) |
| Behcet’s syndrome | 1 (0.4%) |
| Blind | 1 (0.4%) |
| Bursitis | 1 (0.4%) |
| Chiari | 1 (0.4%) |
| Chronic inflammatory response syndrome | 1 (0.4%) |
| Chronic progressive external ophthalmoplegia | 1 (0.4%) |
| Costochondritis | 1 (0.4%) |
| Eating disorder | 1 (0.4%) |
| Long EBV syndrome | 1 (0.4%) |
| Encephalitis | 1 (0.4%) |
| Focal segmental glomerulosclerosis | 1 (0.4%) |
| Histamine intolerance | 1 (0.4%) |
| Insomnia | 1 (0.4%) |
| Long QT syndrome | 1 (0.4%) |
| Lung disease | 1 (0.4%) |
| Pneumothorax | 1 (0.4%) |
| Malabsorption | 1 (0.4%) |
| Mitochondrial disease | 1 (0.4%) |
| Muscular dystrophy | 1 (0.4%) |
| Narcolepsy | 1 (0.4%) |
| Nerve damage | 1 (0.4%) |
| Neuro-disease | 1 (0.4%) |
| Neurodiverse | 1 (0.4%) |
| Night terrors | 1 (0.4%) |
| Obese | 1 (0.4%) |
| Obsessive compulsive disorder | 1 (0.4%) |
| Optic neuritis | 1 (0.4%) |
| Primary biliary cholangitis | 1 (0.4%) |
| Ovarian cyst | 1 (0.4%) |
| Oxalate Sensitivity | 1 (0.4%) |
| Pediatric acute-onset neuropsychiatric syndrome | 1 (0.4%) |
| Pelvic floor dysfunction | 1 (0.4%) |
| Post-COVID syndrome | 1 (0.4%) |
| Primary sclerosing cholangitis | 1 (0.4%) |
| Retinopathy | 1 (0.4%) |
| Scleroderma | 1 (0.4%) |
| Small intestinal bacterial overgrowth | 1 (0.4%) |
| Spinal cord injury | 1 (0.4%) |
| Spinal muscular atrophy | 1 (0.4%) |
| Stiff person syndrome | 1 (0.4%) |
| Stroke | 1 (0.4%) |
| Syringomyelia | 1 (0.4%) |
| Tic disorder | 1 (0.4%) |
| Temporomandibular joint disorder | 1 (0.4%) |
| Vaginismus | 1 (0.4%) |
| Medical Interventions | 57 (20.4%) |
| Mobility aid | 25 (9.0%) |
| Medication | 16 (5.7%) |
| Tube feeding | 11 (3.9%) |
| Alternative therapy | 9 (3.2%) |
| Surgery | 8 (2.9%) |
| Ostomy | 7 (2.5%) |
| IVIG | 5 (1.8%) |
| Psychotherapy | 5 (1.8%) |
| Surgery | 4 (1.4%) |
| Non-invasive testing | 4 (1.4%) |
| Elimination diet | 3 (1.1%) |
| Exercise therapy | 3 (1.1%) |
| Transplant | 3 (1.1%) |
| Blood patch | 2 (0.7%) |
| Infusion | 2 (0.7%) |
| Injection | 2 (0.7%) |
| Insulin pump | 2 (0.7%) |
| Medical alert | 2 (0.7%) |
| Oxygen | 2 (0.7%) |
| PORT | 2 (0.7%) |
| Supplies | 2 (0.7%) |
| TPN | 2 (0.7%) |
| Invasive testing | 1 (0.4%) |
| Catheter | 1 (0.4%) |
| Hearing aids | 1 (0.4%) |
| Lumbar Puncture | 1 (0.4%) |
| Menstrual products | 1 (0.4%) |
| Pap Smear | 1 (0.4%) |
| PICC | 1 (0.4%) |
| PIV | 1 (0.4%) |
| Shunt | 1 (0.4%) |
| Transfusion | 1 (0.4%) |

**Table S2.** Full list of categories and codes with excerpts from post.

| **Category** | **Code and description** | **Excerpt from post** |
| --- | --- | --- |
| **Medical Experience** | **Complications** – Sharing experiences of disease/ treatment complications | “… Operation was successful except for the surprise blood transfusion and accidental cut of my bowel. …” |
|  | **Diagnostic testing** – Experiences with testing | “… Yet another set of invasive inquiries into my body to figure out **why my uterus is a battlefield**. …” |
|  | **Medical encounter** – Referencing specific medical appointments or hospitalization | “… I usually don't get too nervous about doctor appointments. After all, I've had plenty of practice!  But today is my second tilt table test. I've been waiting over a year for this appointment, and now that it's here, my stomach is in knots. …” |
|  | **Negative** – Experiences with healthcare providers/ institutions that are negative | “… Every time I’d leave a doctor’s office after a new autoimmune disease diagnosis, I felt utterly defeated. I felt like my body hated me & was completely against me. 😔 I was told I’d always be overweight, always have pain, always be sick & never have children. FINALLY I said enough is enough! Doctors are just people like you & I and why was I taking a persons opinion as gospel?! …” |
|  | **Positive** – Experiences with healthcare providers/ institutions that are positive | “… My POTs diagnosis changed my life and I am thankful for the doctor who caught the pattern in my symptoms. …” |
|  | **Treatment** – Describing upcoming treatment plans or treatment experiences | “… So, next steps are having 18 more blood tests pending, the anemia will be treated by a hematologist with parenteral iron, esomeprazole may help with the ulcerations, and I'll have to wait a couple weeks for the final [verdict] and treatment with the gastroenterologist. …” |
| **Illness Journey** | **Experience** – Describing experiences related to/ resulted from chronic illnesses | “… A few years ago I could barely make it through a (then) 40 minute class period without needing the bathroom or without being so sick I actually asked a student to run class for me while I sat in a corner and supervised, trash can nearby and doubled over in pain. …” |
|  | **Advocacy/Awareness** – Encouraging self-advocacy or attempting to increase awareness of a disease | “… You know your body best! If you think something is wrong - fight for answers! You might just [surprise] yourself and change YOUR life! ” |
|  | **Illness Misunderstood** – Experiences of their illnesses being misunderstood | “… I've never been one to go to the hospital unless made to. I hate them. The medical gaslighting, the toxic hate for chronic pain patients. The misinformation of Ehlers-Danlos syndrome (of lack of knowledge all together). It's scary. …” |
|  | **Invisible illness** – Focusing on the fact that illnesses exist even though signs/ symptoms are not obvious | “… chronic INVISIBLE illness exists at ALL moments. There is not ONE moment off. However, those moments can look very different from the outside especially to those who lack the knowledge and understanding. …” |
|  | **Advertisements** – Promoting products, services, or activities related to chronic illness | “ Note to the medical community: Every new diagnosis should come with a link to your illness-specific shirt from @spooniesistershop 👚…” |
|  | **Alternative Therapies –** Experiences of alternative therapies for the illnesses | “…I had reached the breaking point and had been watching a few friends around me thriving on this program. It was my turn as a last ditch effort to help myself. I jumped in full body,mind, and soul! Within 4 days my flair subsided and I did not need to get my infusion of steroids. The change happened and for ME it happened rather quickly. …” |
|  | **Illness Impact –** Showing how chronic illnesses have impacted their lives | “…Living with two chronic illnesses makes is so much harder to be flexible, even though I want to be! One reason it is so much harder to be flexible, is because it is more challenging to deal with change when you have a chronic illness to manage. It’s harder to “go with the flow,” harder to pivot and change plans. It’s still possible, just more difficult!  …” |
|  | **Acceptance (Illness Identity) –** Showing self-acceptance of the illnesses | “… there is often a lot of talk about what words are right: "disabled" or "differently abled", person-first or identity-first, which pronouns are valid and which aren't. and yes, there is so much to unravel there -- like how "disabled" is not a bad word, how pronouns are so individual and matter regardless and there are none that are “more/less valid”, how person-first language shouldn't need to exist because we are people, period. there is often a lot of talk about what words are right: "disabled" or "differently abled", person-first or identity-first, which pronouns are valid and which aren't. and yes, there is so much to unravel there -- like how "disabled" is not a bad word, how pronouns are so individual and matter regardless and there are none that are “more/less valid”, how person-first language shouldn't need to exist because we are people, period. …” |
|  | **Enrichment (Illness Identity) –** Sharing how chronic illnesses and related experiences enrich life | “…I will NOT let this disease define me. If anything it drives me to spread awareness and give support to those who also have autoimmune diseases. …” |
|  | **Rejection (Illness Identity) –** Describing efforts/ mindsets of refusing to let chronic illnesses impact life | “…My brain is always in overdrive. Many days I wished to be less smart. Less sharp. Notice less details. Be able to let go of the constant computations and observations my brain makes. Sleep at night instead of existing in a tired body with a brain that won't shut off. …” |
|  | **Engulfment (Illness Identity) –** Experiences of how chronic illnesses overwhelm life and mentality | “…But the spiral that happens each time I’m told “well, there’s nothing of significance that we can find that would be causing this level of pain” on top of everything else is indescribably overwhelming. …” |
|  | **Healing –** Sharing improvements in their illnesses | “…I have not had steroids in over 15 months. Most of my medications are either gone or cut drastically. I am, as they say, “Living my best life!” …” |
|  | **Hope –** Expressing positivity towards the future | “…I don’t think I am nervous at all. More excited than anything. And hopeful. I feel hopeful. …” |
|  | **Self-care –** Sharing practices that improves their health, well-being, and happiness. | “…I stopped a number of times to put my face to the sky and feel the presence of the light and water droplets falling.  To me… this is living. And healing. This is mindfulness in practice. ✨ 🌧 ♥️” |
|  | **Mental Health –** Describing their psychological and emotional well-being. | “…I know I’ve been MIA since I was at Mayo in August but even though Mayo is the greatest hospital it has a huge take on me mentally and I just needed to go ghost for a while and spend much needed time with the people that mean the most to me but now I’m back! Not only am I back on here but I’m back at Mayo again and this time I’m not gonna let it bring me down! …” |
|  | **Sharing Medical Knowledge –** Providing knowledge of the chronic illnesses | “…#Syringomyelia (sear-in-go-my-eelia) is when fluid-filled cysts form in the centre of the spinal cord, hollowing out the cord and compressing/damaging the spinal cord and nerves in that area. This cyst is called a #syrinx (sear-inks). …” |
|  | **COVID –** Referring to COVID, lockdown, or the pandemic | “…Post lockdown life and locks. 💇🏼‍♀️” |
| **Connection** | **People with chronic illness** – Online or in person relationships or messages to other people with chronic illness | “… I [am] extremely [lucky] to have been blessed to have met many others with rare and/or chronic illnesses, however me and @megsmiracles have became super close ! Mainly due to similar diagnosis of #stiffpersonsyndrome , #mthfrgenemutation and many others, but also because of our perseverance to help others not fall into the “broken health care system” …” |
|  | **Support creature** – Relating to support animal or object (i.e. stuffed animal). | “ Being an emotional support frog can be hard work, especially when your human has a chronic illness. …” |
|  | **People without chronic** **illness** – Experiences with and advice to people without chronic illness | “… Don’t be one of those sideline judges. We know most of you don’t understand. But we would love it if you could just try. Try to understand. For one day walk in our shoes. One day see through our eyes. …” |
|  | **Religion** – Sharing religious belief and interaction with their religious god. | “… I find myself a lot of times just breaking down completely when I try to pray. I may only get out the words “Jesus, help me” as the extent of my “prayer”. And for a while I was embarrassed. I thought, how is God supposed to help me if I can’t even give Him a real prayer asking for the things I need? …” |
|  | **Social Media Engagement –** Soliciting interactions with online content. | “…Share this post with a friend or a loved one. Enjoy! 🦋…” |
|  | **Encouraging other online –** Providing encouragement and positivity to social media audience | “…And if you are struggling yourself just keep on going, one moment at a time.  You might be surprised what you find on the other side. …” |
|  | **Keeping it real on social media –** Reminding the differences between social media presentation and reality | “…Just a friendly reminder that most of social media is fake and that the girl in the picture probably doesn’t look like the girl in the picture. …” |
| **Non-illness Content** | **Food –** Sharing food that they made, have, or are interested in. Often describing the food shown in the photos. | “…Not much to look at, but a definite lick your plate clean dish, fine it was! I think that's some variant of chips on the side, no ketchup or mayonnaise, so they might as well not be there! Broccoli how I like it. Soft, salty and soggy. 😂…” |
|  | **Art –** Showing art pieces that they created. | “Some of my art. …” |
|  | **Activities –** Activities in life that are not related to illness, such as reading books, celebrating Halloween, etc. | “So I ran @rocketrace at the weekend and had the best time with the best people. …” |
|  | **Non-medical experience –** Experiences in life that are not related to illness. | “…now only the sofa is coming on Oct 27th ! the rest won't be here till sometime in November 🍑s 🤡's …” |
